# The Influence of Rhenium Content on Helium Desorption Behavior in Tungsten–Rhenium Alloy

**DOI:** 10.3390/ma17112732

**Published:** 2024-06-04

**Authors:** Yongli Liu, Yamin Song, Ye Dong, Te Zhu, Peng Zhang, Lu Wu, Xingzhong Cao, Baoyi Wang

**Affiliations:** 1 The First Sub-Institute, Nuclear Power Institute of China, Chengdu 610005, China; 2 Multi-Disciplinary Research Division, Institute of High Energy Physics, Chinese Academy of Sciences, Beijing 100049, China; 3 Institute of Nuclear and Physical Engineering, Faculty of Electrical Engineering and Information Technology, Slovak University of Technology, Ilkovicova 3, 812 19 Bratislava, Slovakia

**Keywords:** W-Re alloy, positron annihilation spectroscopy, thermal desorption spectroscopy, He-vacancy complexes, rhenium suppression effect

## Abstract

To investigate the influence of different rhenium contents on the helium desorption behavior in tungsten–rhenium alloys, pure tungsten and tungsten–rhenium alloys were irradiated with helium under the same conditions. All irradiated samples were characterized using TDS and DBS techniques. The results indicate that the addition of rhenium can reduce the total helium desorption quantity in tungsten–rhenium alloys and slightly accelerate the reduction in the concentration of vacancy-type defects accompanying helium dissociation. The desorption activation energy of helium is approximately 2 eV at the low-temperature peak (~785 K) and about 4 eV at the high-temperature peak (~1475 K). An increase in rhenium content causes the desorption peak to shift towards higher temperatures (>1473 K), which is attributed to the formation of the stable complex structures between rhenium and vacancies. Besides, the migration of He-vacancy complexes towards traps and dynamic annealing processes both lead to the recovery of vacancy-type defects, resulting in a decrease in the positron annihilation *S* parameters.

## 1. Introduction

One of the primary requirements of interstellar travel is the energy power supply. The space nuclear power system, with efficiency, long lifespan, and tolerance to extreme environments, is considered to be the best solution. Under this assumption, the compact nuclear fusion reactor is thought to be the ultimate ideal space reactor. Tungsten (W) has been studied as a potential material for advanced applications in these systems, leading to a significant production of design-relevant engineering and technological data [1,2,3]. However, one of the most critical challenges is the issue of material radiation resistance. The abundant neutrons generated by a deuterium-tritium nuclear reaction will cause significant radiation damage to materials such as nuclear load-bearing and radiation shielding devices. The bombardment of high-energy neutrons will force lattice atoms to leave their lattice positions and become interstitials which may carry high energy and collide with atoms at other lattice positions, generating cascade collisions. Under the neutron damage effect and cascade collision, a large number of defects such as dislocations, vacancies, vacancy clusters, and voids will appear inside the material. These defects will lead to material hardening, embrittlement, and swelling. As the reactor’s service lifetime increases, defects will continue to accumulate and result in a decline in the mechanical and thermal properties of the material.

During the irradiation, helium (He) will be produced through the fusion reaction. The retention of these inert gas atoms in materials is attributed to the trapping effects of irradiation defects [4]. Since He is not soluble in W, it tends to accumulate at preexisting defects like vacancies or grain boundaries. However, theoretically, it could form mobile interstitial He clusters in W and become immobilized by self-trapping at a “self-created” vacancy by emitting a Frenkel pair consisting of a W vacancy and a self-interstitial W atom in its vicinity (defined as: He*_n_* → He*_n_*V + SIA), leading to the nucleation of gas bubbles. He atoms recombine with each other on cavity surfaces. The sustained high helium fugacity during irradiation elevates gas pressure within both the cavities and bubbles which can be enlarged by dislocation loop punching. Growing bubbles can be interconnected to form new cavities. The high gas pressure inside these cavities, reaching several tens of GPa, is responsible for the development of blisters on the material surface [5,6,7]. He-filled nanovoids (bubbles) are considered to be the precursors to the formation of surface “fuzz”, leading to reduced thermal conductivity and potentially negatively impacting the material’s lifespan. In addition, 14 MeV neutrons can change the elemental composition in tungsten via nuclear transmutations. It is essential to consider the influence of transmutation rhenium (Re) on the microstructure, as well as the mechanical and physical properties of tungsten. It is well known that Re addition can enhance the ductility at low temperatures, improve the high-temperature strength and plasticity, increase the recrystallization temperature, stabilize the grain structure, and enhance the weldability of W. Additionally, W-Re alloy demonstrates superior corrosion resistance compared to pure W. But the high cost of Re—roughly two orders of magnitude greater than W—is a significant drawback. Appropriate addition of Re in W is also an important scientific issue. Studies show that the body-centered cubic structure of the W-Re alloy remains dynamically stable when the content of atomic Re is less than 27%, and key alloy compositions include W-(3~5)Re, W-10Re, and W-(25~26)Re [8]. Alloys containing 5% Re show the optimal balance of hardness and creep strength, while those with over 8% Re display improved workability and significantly better welding properties [2]. Nevertheless, whether Re is intentionally added or naturally produced by neutron transmutation reactions, its presence will inevitably interact with He atoms in W. Therefore, it is of great significance to explore the influence of different Re addition on defects evolution and He retention in W.

Studies have investigated the impact of Re on tungsten’s radiation response. William et al. [9] utilized TEM to investigate W-5%Re, W-11%Re, and W-25% Re in the EBR-II reactor at temperatures ranging from 873 K to 1773 K, with doses between 4.3 and 37 × 10^21^ n·cm^−2^ (E > 0.1 MeV), and found a reduction in void formation due to the irradiation-induced precipitation of Re*_3_*W (c-phase). Hasegawa et al. [10] discovered that the addition of Re can reduce both the size and number density of voids in tungsten. The average positron annihilation lifetime is 115 ps which corresponds to the intrinsic defects in pure W. After neutron irradiation, the long-lifetime component is 494 ps, associated with vacancy clusters of size V*_40+_* in pure W, while the average lifetime is 310 ps, indicating that almost all positrons are trapped at irradiation-induced defects. By comparison, the average lifetime is 138 ps, corresponding to a few defects like dislocations and grain boundaries, in undamaged W5Re. However, in neutron-irradiated W5Re, the long-lifetime component is 468 ps with intensity decreased from 46% to 14%, indicating a much lower density of vacancy clusters in W5Re compared to neutron-irradiated W. Besides, the average lifetime is 173 ps, which is significantly lower than that of irradiated W, suggesting that Re addition suppresses the formation of vacancy-type defects after neutron irradiation. Suzudo et al. [11] investigated the suppression mechanism of rhenium and found a strong mutual attraction between Re atoms and interstitial W atoms. This effectively reduces the mobility of interstitial atoms and enhances the recombination possibility between vacancies and interstitials, thereby suppressing the formation of vacancy-type defects and clusters. In addition, high-density Re clusters were observed in both neutron-irradiated tungsten and W5Re via a three-dimensional atom probe. This is because mutual attraction between Re clusters and radiation-induced defects leads to the generation of complexes of Re clusters and vacancy clusters [12]. Until now, at higher doses of irradiation in many experiments, the generation of Re clusters often accompanies the formation of nanoscale voids in the samples [10,13].

Currently, most studies focus on the influence of Re on neutron irradiation defects, with less discussion on the retention behavior of He atoms within materials and the migration behavior of He-vacancy complexes. Utilizing positron annihilation spectroscopy combined with thermal desorption spectroscopy can effectively elucidate the relationships between solute atoms, vacancy-type defects, and rhenium elements. It is crucial for investigating the radiation resistance properties and the temperature effects on defect evolution in tungsten–rhenium alloys.

## 2. Experimental Details

### 2.1. Sample Preparation

**Figure 1:**
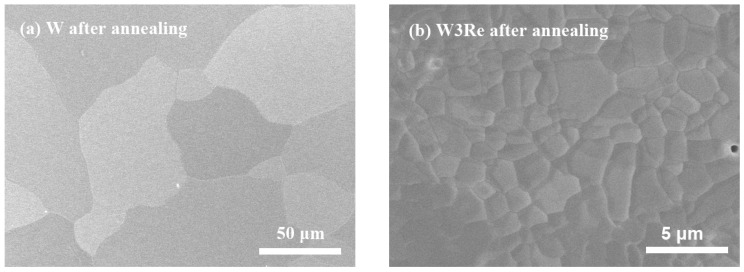

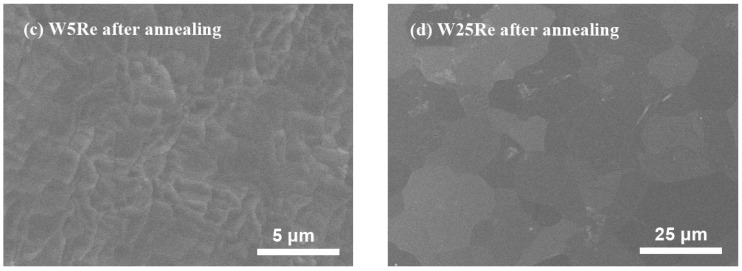
The surface morphology of W, W3Re, W5Re, and W25Re after annealing (1873 K, 1 h) in (**a**–**d**).

Pure W (99.95 wt.%), W3Re (W-3 wt.%Re), W5Re (W-5 wt.%Re), and W25Re (W-25 wt.%Re) alloys of 10 × 10 × 1 mm^3^ were purchased from Xiamen Tungsten Co., Ltd., Xiamen, China (XTC). All samples were mechanically polished with abrasive papers of 30 μm, 20 μm, 15 μm, 10 μm, 8.5 μm, and 3.5 μm grain sizes, respectively, and then were polished with a 0.1 μm colloidal silica suspension to achieve a mirror-like surface. After that, all samples were electrochemically polished in a 2 wt.% NaOH aqueous solution at 273 K to remove contaminants and impurities from the surface and to increase the surface flatness of the samples. After polishing, all samples were thermally annealed at 1873 K for 1 h under a vacuum about 10^−4^ pa to reduce the preparation-induced defects and relieve internal stresses. All samples were fully recrystallized at 1873 K, and the composition of the samples remained constant before and after annealing, with impurity content below 0.05 wt.% [14]. The surface morphology of the annealed sample was characterized by scanning electron microscopy (SEM) in secondary electron (SE) mode, as shown in Figure 1. The average grain size is about 55 μm in annealed W, 2.5 μm in annealed W3Re, 2 μm in annealed W5Re, and 20 μm in annealed W25Re. Obviously, the addition of Re increases the number of grain boundaries per unit area in the W-*x*Re alloy (where ‘*x*’ represents Re content of 3, 5, or 25 wt.%), resulting in at least three times more grain boundaries compared to pure W. Recrystallization restores ductility and reduces hardness by eliminating dislocations and other defects. In pure tungsten, the presence of solute Re atoms leads to segregation at grain boundaries, reducing the grain boundary migration rate through the solute drag effect, thereby inhibiting the grain growth. Additionally, the grain size after recrystallization annealing mainly depends on the pre-deformation and the annealing temperature. Since the annealing temperature is consistent in this study, the initial grain size and deformation state during processing affect the grain size after annealing. If the pre-deformation of W25Re is smaller than that of W3Re and W5Re, the grain size of W25Re is relatively larger after annealing. Nonetheless, the impact of grain boundaries on the results of the subsequent DBS measurement can be ignored. For more details, please refer to our previous work [15].

### 2.2. SRIM Simulation

**Figure 2:**
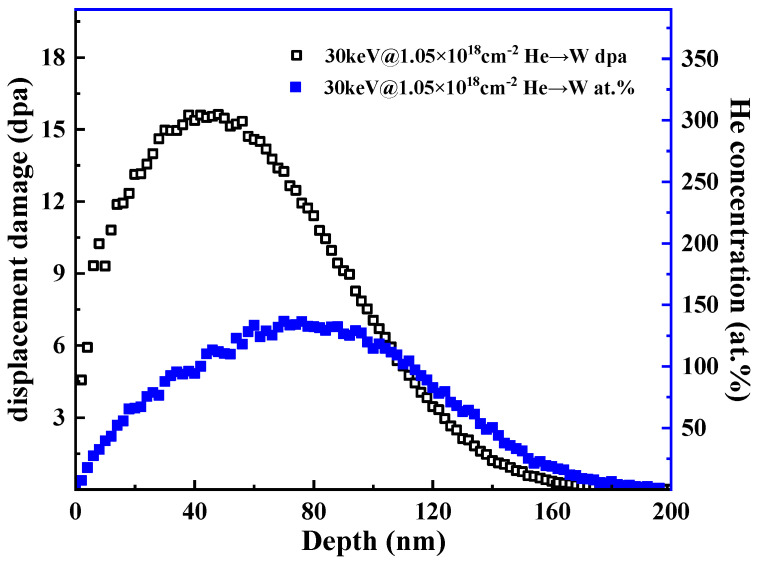
Displacement damage (dpa) and atoms distribution (at.%), respectively, vs. ions implantation depth (nm) under He ions implantation SRIM simulation.

Figure 2 shows the damage profiles and atom concentration profiles in He-irradiated W, W3Re, W5Re, and W25Re using SRIM (version 2008) simulation. The ‘quick KP damage’ mode was selected for both W and W-*x*Re during the simulation. A displacement threshold energy of 90 eV was chosen, along with an incident particle energy of 30 keV and a lattice binding energy of 3 eV for these materials [16,17,18]. The near-surface layer of the sample was damaged to 15 dpa at a depth of 48 nm. The simulation results for W and W-*x*Re alloys exhibited nearly indistinguishable behavior. This can be attributed to the comparable atomic radius for both W and Re. Additionally, the displacement threshold energy (E_d_) depends strongly on the crystallographic direction in which the recoil atoms make replacements and then exchange positions with the matrix atoms along this direction. This process is similar in W and the solid solution tungsten–rhenium alloy in this work [19,20].

### 2.3. Ion Irradiation

He-ion irradiation experiments were conducted on a 50 kV ion implanter at the Institute of High Energy Physics (IHEP). The samples of W (2 pcs), W3Re, W5Re, and W25Re were implanted by 30 keV He^+^ up to a fluence of 1.05 × 10^18^ atom·cm^−2^ at room temperature (RT). The ion beam swept for homogeneous exposure over a 12 × 75 mm^2^ implantation area. All samples were mounted on a stainless steel holder with water cooling to remove the excess heat generated by the irradiation process.

### 2.4. Thermal Desorption

Thermal Desorption Spectroscopy (TDS) is a technique used to investigate defects in metals, particularly those created by ion implantation, and their thermal stability [21]. He-ion irradiation creates a multitude of defects such as vacancies and self-interstitials. He atoms become trapped at these defects or pre-existing defects. When the sample is heated, the helium dissociates from the defects or migrates as complexes and then is released from the surface, resulting in desorption peaks that correspond to the release from different defects present in the sample. The temperature at which each peak occurs is indicative of the type of defect, with higher temperature peaks typically corresponding to defects with higher binding energies. By measuring the helium released rate as a function of temperature with a constant heating rate and analyzing the shape and location of these peaks, information about the nature and distribution of defects in the material can be obtained.

In this work, both W and W-*x*Re alloys were first irradiated with He ions at RT and then analyzed by TDS to examine helium desorption and determine the kinetic parameters governing the release of helium from the irradiation-induced defects. A ceramic heater was used to heat the samples at a constant rate of 1 K/s, starting from RT and reaching a maximum temperature of 1573 K. A K-type thermocouple was clamped to the surface of the sample to measure the temperature, which was controlled by a Proportion Integration Differentiation (PID) controller with a feedback loop. The uncertainty of temperature measurement was ±2 K. However, there was an exception for sample W-1#, which was heated up to 1133 K and then stopped. The thermal desorption experimental parameters for all samples are listed in Table 1. Different gas molecules were released and ionized during the TDS and then monitored by a compact quadrupole mass spectrometer (QMS) of Dycor Dymaxion DM200M. A mass spectrometer enables the identification of the masses of individual atoms and molecules that have been ionized from a given sample. This technique is distinctive as it provides a fingerprint identification for the structural and chemical properties of these molecules. Before the experiment, a standard gas leak with He (33.34%), Ar (33.33%), and N_2_ (33.33%) with an inaccuracy smaller than 6.17% was employed to calibrate the QMS. The calculated desorption sensitivity coefficient of helium is 7.1878 × 10^21^ s^−1^A^−1^. It has been established that the release of helium from highly pressurized bubbles formed during irradiation occurs at a lower temperature than the temperature at which remaining nano-cavities dissociate [22]. Additionally, TDS provided quantitative information, such as the population of defects and the amount of helium remaining in the samples after annealing. Overall, these findings demonstrate the power of TDS as a tool for investigating defects in metals and provide valuable insights into the behavior of helium bubbles during annealing.

## 3. Characterization Methods

### 3.1. Slow Positron Beam (SPB)

^22^Na is commonly utilized in the laboratory as a positron source, primarily due to its moderately long half-life of approximately 2.6 years and the moderate average energy of its decayed positrons, which is around 260 keV. This source continuously emits positrons with a maximum energy of 545 keV, comprising about 90.2% of its emission spectrum. Empirical formulas, such as $Z\left(E\right)=40\times E^{1.6}/\rho$, enable the calculation of the average penetration depth (Z) of positrons with a specific energy (E) in a given material with a mass density of ρ (g/cm^3^). For the study of shallow irradiation regions (<1 μm), it is crucial to reduce the energy of conventional positrons, as their characteristic range in pure tungsten is approximately 10~50 μm, which is not ideal for the current research objectives. To address this, a tungsten mesh with a relatively large negative-function energy value (approximately −2.1 eV) is employed as a moderator in the slow positron beam facility at IHEP. This allows for continuous adjustment of the positron beam energy within the range of 0.18 to 20 keV under a vacuum of 10^−7^ Pa. The radioactivity of the ^22^Na positron source is estimated to be around 9 mCi.

### 3.2. Doppler Broadening Spectroscopy of Slow Positron Beam (DBS)

The positron-electron annihilation process strictly adheres to the law of momentum conservation. As the annihilated electron possesses a certain momentum, it induces an evident Doppler energy shift relative to the energy of the annihilation photon ($m_{e}c^{2}=0.511\;MeV$). The two photons with identical energy are emitted in opposite directions. By capturing a substantial number (>10^6^) of annihilation events through a single HPGe detector with a resolution of about 1~1.5 keV (at 511 keV), a symmetric peak distribution is obtained, centered at a photon energy of 0.511 MeV. The shape of the peak, determined by the momentum of the annihilated electron, is defined by two parameters, namely the *S* parameter and *W* parameter. The *S* parameter signifies positron annihilation with a valence electron of smaller momentum in the outer orbital layer of the target atom. The *W* parameter represents the interaction between the positron and a high-momentum electron, commonly known as a core electron, within the inner orbital layer of the target atom.

## 4. Results and Discussion

### 4.1. Thermal Desorption Spectra from the He-Irradiated W-xRe Alloys

**Figure 3:**
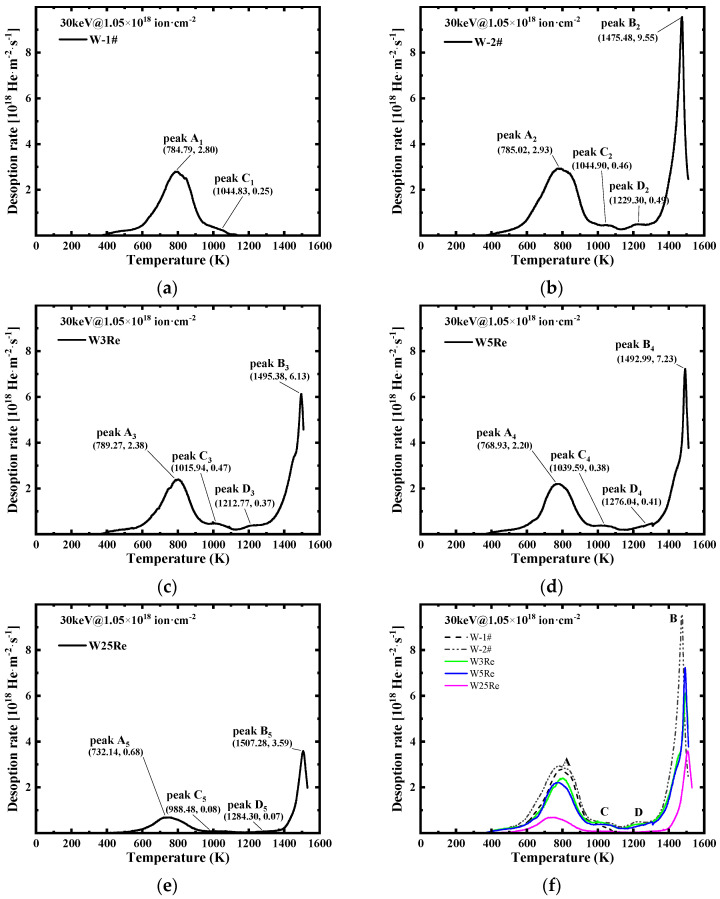
Thermal desorption spectrum of helium in W-1# (**a**), W-2# (**b**), W3Re (**c**), W5Re (**d**), and W25Re (**e**) after He-ion irradiation at RT. Desorption spectra of all samples were plotted on a single graph (**f**), where A and D represent the main peaks around 785 K and 1475 K, respectively, and B and C represent the shoulder peaks around 1044 K and 1229 K.

After helium implantation experiments and DBS measurements, TDS was performed at IHEP. Figure 3 shows TDS spectra of He released from W-1# (a), W-2# (b), W3Re (c), W5Re (d), and W25Re (e) after 30 keV He irradiations with the same fluence of 1.05 × 10^18^ atom·cm^−2^ at RT. During the experiment, the desorbed helium atoms were ionized in the ion source and accelerated into the QMS system. Helium was mainly desorbed as monoatomic molecules, and the signal intensity of HD and H_2_O molecules was lower than that of He by an order of magnitude or more.

As shown in Figure 3a, when pure tungsten is heated to 1173 K and maintained for 30 min, a distinctive peak emerges at 785 K, and a shoulder peak appears at 1044 K. For comparison, when pure tungsten is heated from RT to 1573 K, similar peaks are observed, with an additional shoulder peak at 1229 K and a prominent peak at 1475 K, as illustrated in Figure 3b. Each of these peaks seems to consist of multiple narrower desorption peaks. The first-order desorption peaks, representing the release of a single helium atom from a trapping site, exhibit Full Width at Half Maximum (FWHM) values of 0.07 times the peak temperature (*T_m_*), i.e., 55 K and 103 K for the peaks at 785 K and 1475 K, respectively. Therefore, the analysis should take two groups of peaks into consideration: one centered around 785 K at low temperatures and another centered around 1475 K at high temperatures. As drawn in Figure 3c–e, with an increase in the content of Re addition, the low-temperature group (785 K) shifts towards lower temperatures, while the high-temperature group (1475 K) shifts towards higher temperatures. Due to the similar rhenium content in W3Re and W5Re, the distinction in desorption peak positions cannot be distinguished. However, in the case of W25Re, a significant decrease in total desorption is observed, as shown in Figure 3e. The population of desorption helium per unit area and its ratio to the implanted helium are summarized in Table 2. The population of desorption helium was calculated by integration of the He desorption rate. Under 30 keV and 10^18^ He·cm^−2^ irradiation, the helium population decreases as the rhenium content increases. When pure tungsten is heated to 1573 K, the desorption quantity is 0.53 × 10^18^ He·cm^−2^. The desorption difference between W and W25Re is just by a factor of 2.68, the latter is close to that of W-1#, approximately 0.2 × 10^18^ He·cm^−2^. In general, the ratio desorption/implanted is not close to 100% in this study. Nearly all desorption fractions are several tens of a percentage lower than unity. This is because the experiment relied heavily on discrete datasets, neglecting the continuous summing process. Furthermore, the amount of backscattering fraction is high for low-energy ion implantation, suggesting that helium retention did not reach saturation in this study [22].

The concentration of trapped He, $C_{He-trap}$ is expressed as follows:

$$C_{He-trap}=\sum_{i}C_{t,i}\theta_{t,i}$$

In first-principle calculations, various types of defects in W or W-Re alloy provide multiple accommodation sites for trapped He atoms. Therefore, $C_{t,i}$ is the concentration of accommodation site i for trapped He atoms in the defect and $\theta_{t,i}$ ($\theta_{t,i}\leq 1$) is the fraction of the defect’s accommodation site i occupied by a He atom. Each trapped He atom is characterized by distinct binding energy. We need to clarify that $C_{t,i}$ is not the concentration of defects but maintains a proportional relationship with the radiation-induced defects. As only one He atom can be embedded in each accommodation site, the occupation probability, denoted as $\theta_{t,i}$, is subject to a local trapping-detrapping equilibrium at different temperatures T, which is expressed as follows:

$$\frac{\theta_{t,i}}{1-\theta_{t,i}}=\frac{\theta_{L}}{1-\theta_{L}}exp(\frac{E_{bin,i}}{kT})$$

Here, $\theta_{L}$ represents the fraction of occupied interstitial sites, which is determined by the solubility of He and the temperature under He ion irradiation. $E_{bin,i}$ denotes the binding energy between accommodation site i and a trapped He atom in an interstitial site. k is the Boltzmann constant. It is reasonable to assume that the solubility of He atoms in W is comparable with W-*x*Re, as well as the diffusivity. The TDS spectra of He released from W and W-*x*Re, as shown in Figure 3f, exhibit similar main peaks in the same temperature range, signifying that the values of $E_{bin}$ are comparable among these samples. Consequently, the concentration of trapped He is determined by the concentration of accommodation sites within defects. The variation in $C_{t,i}$ can account for differences in the thermal desorption spectra height under identical irradiation conditions. Obviously, the concentration of irradiation-induced vacancy-type defects acting as trapping sites in W25Re is significantly lower than that in W, as well as W3Re and W5Re. This to some extent proves that Re addition can reduce the concentration of vacancy-type defect. This mechanism can be explained by the mixed dumbbell structure formed between the Re atom and interstitial W atom in tungsten. The low rotation energy barrier of the W–Re dumbbell allows Re interstitials to exhibit three-dimensional motion which has a higher probability of accelerating the recombination between vacancy and interstitials [11]. 

The simple first order detrapping mechanisms were applied in this study with attempt frequencies (ν) of an order of the Debye frequency ($10^{13}\;s^{-1}$). The corresponding desorption activation energy of the two groups of peaks can be roughly estimated by the formula given as follows:

$$\frac{E_{d}}{kT_{p}^{2}}=\frac{\nu }{\beta }exp(-\frac{E_{d}}{kT_{p}})$$

The deformation of this formula is given as follows:

$$2lnT_{p}-ln\beta =(\frac{E_{d}}{k})\frac{1}{T_{p}}+ln\frac{E_{d}}{k\nu }$$

where $E_{d}$ is desorption activation energy (the dissociation energy in some literature), k is the Boltzmann constant, β is the heating rate (=1 K/s), and $T_{p}$ is the absolute peak maximum temperature. There is a linear relationship between ($2lnT_{p}-ln\beta$) and $\frac{1}{T_{p}}$. By measuring the slope of the line, the activation energy $E_{d}$ can be obtained, which is shown in Table 3. 

The $E_{d}$ is about 2 eV found in the low-temperature group of peaks at 785 K and about 4 eV in high-temperature group at 1475 K in damaged W-2#, W3Re, W5Re, and W25Re. The knock-on energy threshold for He-induced displacement damage in W is about 500 eV, meaning that the 30 keV He ion irradiation in this work is sufficient to introduce a large number of preexistent vacancies or vacancy clusters in the material [23]. These defects are crucial for the retention of He in W. According to the difference in peak heights observed in the two peak groups in Figure 3f, one can assume that the low-temperature group is due to the dissociation of He from the small-size He-vacancy complexes (He*_n_*V*_m_*) with a large *n*/*m* ratio or clusters of interstitial He (He_n_) with *n* ≥ 2, while the high-temperature group is attributed to the detrapping from the He atom decorated with large-size vacancy clusters (He*_n_*V*_m_*) with small *n*/*m* ratios or even voids (m ≥ 3) [22,24]. Studies have shown that high-flux irradiation increases He retention in W due to the higher density of He*_n_* clusters (*n* ≥ 6), making the He self-trapping mechanism (He*_n_* → He*_n_*V) more likely [23,25]. Dunand et al. [26] observed two He desorption peaks around 950 K and 1700 K in a single crystal W(110) sample irradiated by He ions with an energy of 130 eV (without pre-existing vacancies) at a flux of 0.7 × 10^17^ m^−2^·s^−1^. These peaks correspond closely to the dissociation energies of self-trapped He*_n_*V clusters. As the flux continues to increase, the trap mutation mechanism (defined as: He*_n_*V*_m_* → He*_n_*V*_m_*_+1_ + SIA) will be triggered, leading to the appearance of He desorption peaks above 1800 K and a shoulder at 1900 K. When the ion energy exceeds the threshold for vacancy production, the pre-existing defects induced by irradiation are responsible for the prominent He desorption peak around ~1100 K. These defects serve as efficient nucleation sites for the He-vacancy clusters such as He*_n_*V*_m_* with large *n*/*m* ratio, compared to self-trapping of He*_n_* clusters.

During implantation, helium atoms become trapped in shallow structural defects (e.g., single vacancies, vacancy/atom-type dislocations), forming He*_n_*V*_m_* clusters with large *n*/*m* ratio, as well as internal bubbles, and surface blisters whose $E_{d}$ in pure tungsten is approximately 2.23 eV. Nobuta et al. [4] found that in undamaged tungsten, the majority of deuterium (D) is released between 800~1200 K, primarily trapped in intrinsic defect sites like dislocations and vacancies. As the dose accumulates, more helium atoms are prone to be captured by irradiation-induced large-size vacancy clusters or voids. And in the subsequent annealing, the enhanced diffusion of defects plays an important role, further promoting the nucleation, growth, migration, and aggregation of defect clusters, such as bi-vacancies and vacancy clusters. Helium atoms become trapped near such defects of small *n*/*m* ratio. In pure tungsten, the $E_{d}$ of helium can reaches 4.28 eV. Furthermore, a shift in the two peaks is observed in different tungsten–rhenium alloys. With increasing rhenium content, a slight decrease in the $E_{d}$ of shallow structural defects is observed, reaching 2.08 eV in W25Re, indicating a change in the nature or size of defects. For example, smaller-size defects or defects with higher coordination numbers of He atoms may require lower He dissociation energy. The addition of rhenium suppresses the growth of size or concentration of such vacancy defects. In the high-temperature group, the $E_{d}$ in W25Re is 4.37 eV, slightly higher than in pure tungsten, indicating a more complex structure of large-size defects. Through density functional theory (DFT) calculations [23], it has been found that the binding energy between Re and He clusters varies from 0.03 eV for Re-He*_1_* to 0.72 eV for Re-He*_4_*. This significant difference can be attributed to the lattice distortion caused by He clusters, which reduces the electron density at the He occupation sites and induces neighboring Re atoms to transition from substitutional sites to interstitial sites. Due to the attraction between Re and He clusters, there is a strong pinning effect of Re on the migration of He clusters. The addition of Re effectively reduces the mobility of He clusters in W. Consequently, the positive binding energy among Re atoms, He clusters, and neighboring point defects such as vacancies and self-interstitial atoms makes it easy to form Re-He*_n_*-vacancy complexes. In addition, the Ostwald ripening process, where larger vacancy clusters grow at the expense of smaller ones, will promote the growth of vacancy-type clusters in these complex structures [22], thereby forming more stable defects and leading to a shift of the 1475 K desorption peak towards higher temperatures.

Some experiments have detected He desorption peaks below 700 K [26]. Iwakiri et al. [27] proposed the impurity-assisted self-trapping mechanism, wherein He aggregates around a bulk impurity. Upon ejection of the impurity, the He cluster is immobilized at the created vacancy. Kornelsen et al. [28] believed that the presence of surface impurities will probably lead to the appearance of He desorption peaks below 800 K [28]. In addition, the spatial distribution of He*_n_*V*_m_* within the sample also influences the He dissociation energy, which tends to diminish as He*_n_*V*_m_* clusters get closer to the surface. The *n*/*m* ratio increases to 20 with a He dissociation energy of approximately 1.8 eV (~640 K). From Figure 3, it can be seen that none of the samples in this work exhibit these low-temperature He desorption peaks.

### 4.2. Near-Surface Damage Depth Profiles from the He-Irradiated W-xRe Alloys

**Figure 4:**
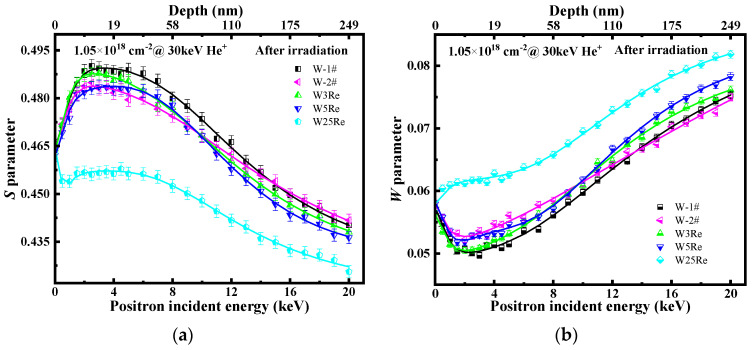
(**a**) Low-momentum annihilation fraction (*S* parameter) and (**b**) high-momentum annihilation fraction (*W* parameter) vs. positron energy of He-ion irradiated W-1#, W-2#, W3Re, W5Re, and W25Re before thermal desorption experiment.

All irradiated samples were characterized by DBS before TDS experiments. The *S* parameter and *W* parameter vs. depth are presented in Figure 4a,b. From Figure 4a, the surface *S* parameters are significantly higher than those of the matrix in W and W-*x*Re alloy samples post-irradiation. This is attributed to surface effects of samples where ion bombardment causes lattice distortions on the surface, leading to production of structural defects that anomalously attract positrons. In W, W3Re, and W5Re, the *S* parameter reaches its maximum value between 9~40 nm and is almost consistent within the error bar. The *S* parameter peak position (~10 nm) is shallower than the SRIM calculation results (~48 nm), which is due to surface effects and defect migration towards the surface. Additionally, defect distribution extends well beyond a depth of 160 nm (the matrix), indicating the diffusivity of vacancy-type defects. Notably, the level of irradiated-induced defect in W25Re alloy are markedly lower than that in W, W3Re, and W5Re. Studies suggest that the complex structures formed between Re and W atoms suppress the concentration accumulation of defects within the depth range, which is consistent with TDS results. Recent calculations have shown that solute segregation occurs in the form of voids decorated by Re atoms, even in the low Re concentrations range, and these vacancy-Re clusters remain stable at temperatures above 800 K [29]. A single Re atom can significantly enhance the binding energies between two or three vacancies [30]. Some experiment results signify that significant voids are present in the bulk of all W-Re alloys [31]. Hasegawa et al. [10] found that the addition of Re reduced both the size and number densities of voids formed in tungsten. This suppression effect is mainly due to the inhibition of Re on the formation process of vacancy type defects. The W-Re dumbbell’s low rotation energy barrier enables Re interstitials to move three-dimensionally, increasing the possibility of vacancy-interstitial recombination [11]. *W* parameter distribution vs. depth exhibit a mirror symmetric distribution with the *S* parameter. The variation of the *W* parameter is often related to the chemical environment at the defect site, which is not the topic of discussion in this paper.

**Figure 5:**
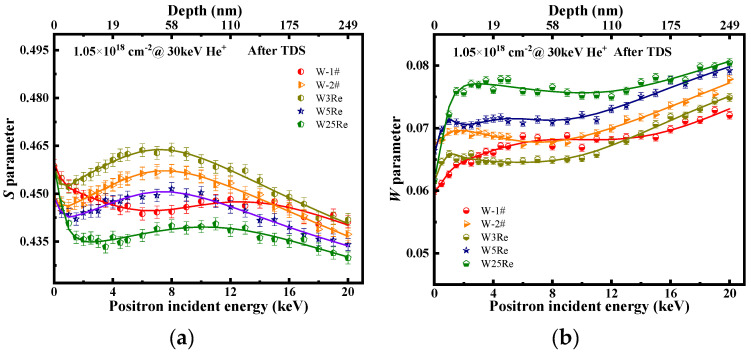
(**a**) Low-momentum annihilation fraction (*S* parameter) and (**b**) high-momentum annihilation fraction (*W* parameter) vs. positron energy of He-irradiated W-1#, W-2#, W3Re, W5Re, and W25Re after TDS experiment.

After desorption, the samples were naturally cooled down to RT under vacuum and then characterized by DBS. The depth distributions of the *S* parameter and *W* parameter are shown in Figure 5a,b, respectively. For W-2#, the maximum *S* parameter decreased significantly from 0.482 (corresponding to the depth of 10 nm before desorption) to 0.456 (50 nm). The decrease in the *S* parameter indicates a reduced probability of positron annihilation with conduction electrons at vacancy-type defects, suggesting a significant decrease in concentration of vacancy-type defect within this depth range. 

On the one hand, the dynamic annealing during the thermal desorption process leads to the migration and recovery of point defects, especially single vacancies. Recovery stages of specific types of defects and the corresponding temperatures in tungsten have been studied in ref. [32]. The first stage is below 100 K, where free interstitials become mobile, migrating towards vacancies and other defect sinks [33,34]. Larger defect structures such as dislocation networks or grain boundaries serve as traps for interstitials at higher doses [35,36,37]. A second stage between 100 K and 520 K sees interstitials released from traps like displacement cascades [36]. The third stage, at approximately 520 K, focuses on the defect recovery due to the migration of mono-vacancies [32,34]. It is observed that the mono-vacancies begin to be mobile at 550 K towards sinks, recombining with self-interstitials or combining into larger defects [34,38]. The fourth stage, around 800 K, is about the defect recovery which is due to the mobility of larger defects such as vacancy-impurity complexes and di-vacancies [34]. The fifth stage, around 1100 K, is attributed to the formation of voids formation and breaking down of defect clusters [33]. Therefore, thermal annealing in this study from RT to 1573 K will cover these five recovery stages, resulting in a significant decrease in defect concentration. Research indicates that tungsten exposed to doses greater than 3.2 × 10^−2^ dpa results in approximately 5% defect recovery due to defect release from traps after low-temperature annealing to 473 K and about 70% defect recovery after annealing to 1073 K. In particular, the most substantial recovery, about 50%, was observed during annealing at 673 K, highlighting significant healing between 473 K and 673 K [32,35]. 

On the other hand, the dynamic annealing process decreases the concentration of large-sized defects at He dissociation sites. During the irradiation, He atoms implanted into tungsten were strongly trapped by different types of defects like vacancies, grain boundaries, dislocations, and voids. The calculation result shows that the energy of a single He atom trapped by a mono-vacancy is about 4.5 eV, which easily forms the structure of He-vacancy complexes [39,40]. The size of He-vacancy complexes will be increased by trapping additional He atoms. At RT irradiation, He-vacancy complexes exhibit limited diffusion, confining He trapping by grain boundaries mainly to interstitial He diffusion. At the same time, He atoms can aggregate at interstitial sites and form clusters [41]. During the TDS experiment, as temperatures rise to 1573 K, some He-vacancy complexes within the matrix begin to diffuse and coalesce to form bubbles or diffuse to the surface or the grain boundaries. The enhanced trapping of interstitial He by grain boundaries and the increased diffusion of He-vacancy complexes towards these boundaries can be attributed to the high sink strength, characterized by a dense grain boundary network of these samples, and the short diffusion paths for the He-vacancy complexes [42]. Therefore, as He-vacancy complexes diffuse to the sample surface or grain boundaries and undergo helium desorption, the concentration of large defects in sample decreases, leading to a significant decrease in the *S* parameter.

In addition, due to the insufficient holding time of the high-temperature desorption at 1573 K, the medium-sized defect clusters formed from the breakdown of the defect clusters have not yet been released by He dissociation. These defects still exist in the sample, as evidenced by the peak shape of broad and shallow observed in the depth distribution of the *S* parameter between 12 and 141 nm in Figure 5a. After desorption, the *S* parameter decreases notably with increasing Re content. However, W3Re shows an upward trend, a phenomenon that has been observed in previous studies as well [15]. It can be confirmed now that 3% Re content does not strongly inhibit the formation of vacancy-type defects, which is due to 3% Re addition enhancing the annihilation probability of positrons at He-vacancy defect decorated with Re atoms. Similarly, a study has shown that Re addition can enhances the positron annihilation probability at the site of hydrogen-trapping vacancy defects [43].

High-temperature annealing enhances the Re effect, showing that Re content (≥5%) can effectively suppress the size and concentration of vacancy-type defects in tungsten. Re atoms interact strongly with defects, forming structures of Re-vacancy complexes. The probability of positron annihilation with the low momentum electron at such defects is small than the matrix, leading to a decrease in the *S* parameter with increasing Re content.

**Figure 6:**
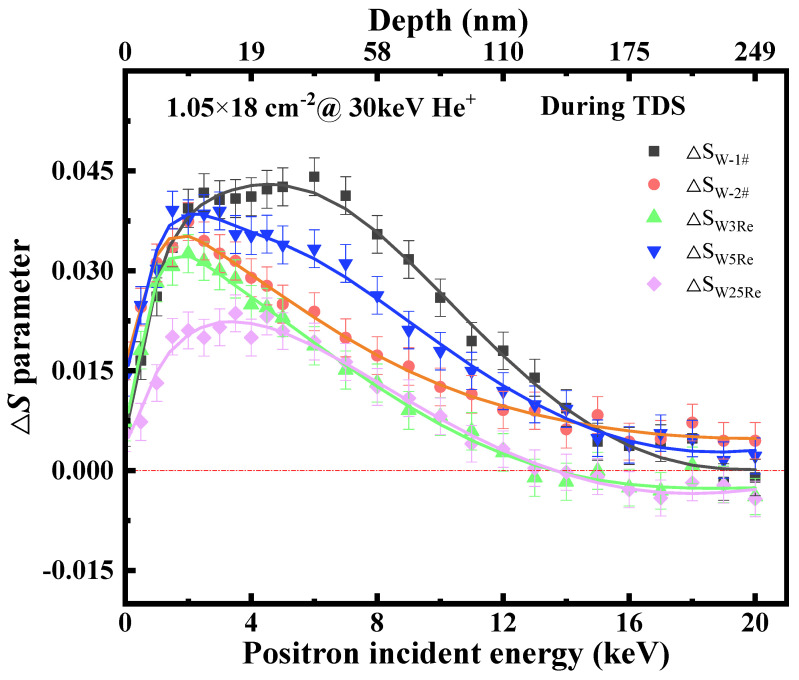
Δ*S* parameter (the difference in the *S* parameter before and after desorption.) vs. positron energy of He-irradiated W-1#, W-2#, W3Re, W5Re, and W25Re.

The relationship between the Δ*S* parameter (Δ*S = S*_2_ *− S*_1_) and depth is shown in Figure 6. *S*_2_ is the *S* parameter of samples before TDS, and *S*_1_ is the *S* parameter of samples after TDS. For all samples, the Δ*S* parameter has an almost positive value. This indicates a decrease in the annihilation probability of positrons with low-momentum electrons at the vacancy-type defect locations, implying the recovery of vacancy-type defects during the TDS process. It is obvious that there is significant defect recovery within the depth range from the surface to 175 nm during the annealing from RT to 1573 K. Beyond a depth of 175 nm, the Δ*S* parameter approaches zero within the error range, indicating that the annihilation probability of positrons is almost consistent before and after TDS. This does not mean that there are no defects within this range. As observed in Figure 4 and Figure 5, when the depth is deeper than 175 nm, the *S* parameter for all samples continues to decrease, but it does not reach the matrix defect level until 249 nm (*S_matrix_* ≈ 0.43, please refer to [16]). Annealing at 1573 K for a relatively short duration results in the existence of a large number of vacancy-type defects beyond the depth of 175 nm. Only after recrystallization annealing at 1873 K do the samples reach the matrix level at depths deeper than 212 nm. Such vacancy-type defects are most probably due to irradiation-induced vacancy clusters, which continuously merge or coarsen during migration and have not yet been released at the sink. Therefore, the *S* parameter shows little change before and after TDS, yet is still higher than the defect level of the matrix.

Moreover, in tungsten–rhenium alloys, the differences in Δ*S* parameter distribution are minimal (just about 0.015), indicating a similar decrease in the concentration of vacancy-type defects in samples with different Re contents. However, as seen in Table 2, the amount of He desorption decreases with an increase in Re content, which suggests that the addition of Re enhances the migration of He-vacancy complexes towards sinks and their release at these sinks. For instance, among the samples subjected to TDS at 1573 K, the amount of He dissociated from W25Re is the least. However, the recovery of vacancy-type defects is comparable to that of W-2#. This could be attributed to the influence of Re, which makes He-vacancy defects more prone to attracting other vacancy defects, leading to the formation of larger He-vacancy clusters. Subsequently, during helium clusters’ migration towards and release at the sinks, it accelerates the reduction in the concentration of vacancy-type defects in the samples.

For W-1# (heated to 1133 K and then stopped), the recovery of vacancy-type defects within the depth range of <175 nm is stronger than that of W-2# (heated to 1573 K and then stopped). However, in Figure 3f, the total amount of He dissociated from W-2# is higher. The two results seem contradictory. One can speculate that the heating process from 1133 K to 1573 K not only facilitates the release of He-vacancy complexes at the sinks but also promotes the diffusion of medium-sized defect clusters towards shallower surfaces, thus compensating for the defect concentration between 10~150 nm. After stopping the heating at 1133 K, these larger vacancy clusters cease to diffuse, resulting in a gap layer in the defect depth distribution of W-1#, manifested as the “valley” shape from 10 to 110 nm in Figure 5a.

### 4.3. The S-W Distributions of the He-Irradiated W-xRe Alloys

**Figure 7:**
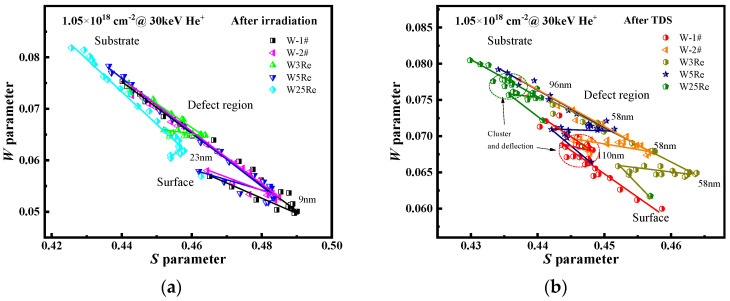
*S* parameter vs. *W* parameter in He-irradiated W-1#, W-2#, W3Re, W5Re, and W25Re before (**a**) and after (**b**) TDS experiment, respectively.

The *S-W* trajectory is always used to evaluate the types of vacancy-type defects in material. The distribution of (*S*, *W*) data along lines with similar slopes implies the same types of defects or He*_n_*V*_m_* complexes of different sizes with the same ratios of *n*/*m* in samples. However, deviations in the *S-W* trajectory always indicate changes in defect types. The two plots in Figure 7a,b can be roughly divided into three regions: surface, substrate, and the irradiation defect region. It can be observed that the (*S*, *W*) distribution is broad before desorption in Figure 7a. The trajectories of W-2#, W3Re, and W5Re have the same slopes, indicating that similar types of defects are dominant in these samples. The slope of W25Re deviates from that of W-2#, suggesting a possible change in defect types. This deviation may be attributed to the involvement of Re atoms in the formation of He-vacancy complexes. After desorption, (*S*, *W*) distribution gradually narrows, indicating that the thermal desorption process promotes a more uniform distribution of defects. This is due to the high temperature causing defect annealing and the migration of vacancy-type defects in different depth layers. Importantly, the (*S*, *W*) data of W-2#, W3Re, W5Re, and W25Re distribute more narrowly and exhibit turning points with increasing Re content, although their slopes are similar, suggesting that the addition of rhenium mainly suppresses the concentration of vacancy-type defects. A study [4] shows that adding Re into the W5Re alloy inhibited the formation of trap sites induced by He ion irradiation, leading to smaller gas atom retention in the W5Re alloy than pure tungsten. The mechanism of Re addition suppressing gas atom retention is attributed to the inhibition of vacancy-type defect formation [44]. In the case of W25Re, clustering and deflection of (*S*, *W*) data occur at 96 nm, indicating the formation of more complex defect structures within this depth range. A similar situation in W-1# is observed at 110 nm, which is due to the evolution of irradiation defects, including migration, aggregation, and coarsening, producing the complex defect structures. Meanwhile, the *S-W* trajectory of W-1# differs in slope from the other samples, being closer to the slope of the surface defects in W25Re. This suggests that the defect type of W-1# is similar to the near-surface defect type of W25Re. Furthermore, the defect concentration of W-1# is relatively higher than that in W25Re, with (*S*, *W*) data distributed in the high *S*, low *W* region.

## 5. Conclusions

All samples irradiated by He ions with energy of 30 keV and fluence of 1.05 × 10^18^ atom·cm^−2^ at RT were characterized by TDS and DBS experiments. It can be determined that various Re contents in tungsten will have a certain degree of influence on the desorption behavior of helium, which can be summarized as follows:

1. Increasing rhenium content will reduce the total helium desorption population in W-*x*Re alloys. This is due to the formation of a mixed dumbbell structure of Re atoms and W atoms. The three-dimensional migration of these structures will increase the probability of recombination between interstitials and vacancies, thereby reducing the concentration of vacancy type defects and suppressing the retention of helium, which is strongly attracted by defects.
2. The desorption activation energy of helium in all samples is about 2 eV at the low-temperature peak (785 K) and about 4 eV at high-temperature peak (1475 K). The former is attributed to the dissociation of helium from small-sized He-vacancy complexes or interstitial He clusters, while the latter is attributed to the desorption of helium from large-sized vacancy clusters or even voids.
3. With the increase in rhenium content, the high-temperature peak at 1475 K shifts towards higher temperatures. This is because the complex structures formed by the strong attraction among Re, He clusters and vacancies promote the growth of vacancy type clusters to more stable defects during the Ostwald ripening process.
4. Thermal desorption will lead to a significant decrease in *S* parameters, which originates from two main factors. Firstly, the migration of He-vacancy complex defects towards sinks during thermal desorption process leads to recovery of defects in these low-potential regions. Secondly, the migration and recovery of single vacancies during dynamic annealing, as single vacancies serve as the nucleation of vacancy clusters of different sizes.
5. The addition of rhenium slightly accelerates the reduction in the concentration of vacancy-type defects accompanying helium dissociation. However, the latter is primarily determined by the annealing temperature and annealing duration.

## Figures and Tables

**Table 1:** Details of thermal desorption experiments.

Sample	Vacuum (Torr)	Initial Temperature (K)	Heating Rate (1 K·s^−1^)	Ending Temperature (K)	Holding Time (min)
W-1#	1 × 10^−8^	293	1	1133	30
W-2#	1 × 10^−8^	293	1	1573	2
W3Re	1 × 10^−8^	293	1	1573	2
W5Re	1 × 10^−8^	293	1	1573	2
W25Re	1 × 10^−8^	293	1	1573	2

The symbol # represents the number of samples.

**Table 2:** Population of desoption helium [10^18^ cm^−2^] and the ratio to implanted helium [10^18^ cm^−2^].

W-1#	Ratio	W-2#	Ratio	W3Re	Ratio	W5Re	Ratio	W25Re	Ratio
0.20	0.19	0.53	0.51	0.40	0.38	0.40	0.38	0.20	0.19

**Table 3:** The desorption peak temperature ($T_{p}$) and the desorption activation energies ($E_{d}$) of He atoms in damaged W-1#, W-2#, W3Re, W5Re, and W25Re.

Traps *	W-1#		W-2#		W3Re		W5Re		W25Re	
	*T_p_* (K)	*E_d_* (eV)	*T_p_* (K)	*E_d_* (eV)	*T_p_* (K)	*E_d_* (eV)	*T_p_* (K)	*E_d_* (eV)	*T_p_* (K)	*E_d_* (eV)
He*_n_*V*_m_* (high *n*/*m* ratio) or He_n_ clusters	784.79	2.23	782.58	2.23	789.27	2.25	768.93	2.19	732.14	2.08
He*_n_*V*_m_* (small *n*/*m* ratio) or voids	/	/	1475.96	4.28	1495.38	4.34	1492.99	4.33	1507.28	4.37

* Traps are main sites retaining helium.

## Data Availability

The original contributions presented in the study are included in the article, further inquiries can be directed to the corresponding authors.
